# A case of mucinous cystic neoplasm of the liver: a case report

**DOI:** 10.1186/s40792-014-0007-z

**Published:** 2015-01-30

**Authors:** Yusuke Nakayama, Yuichiro Kato, Satoshi Okubo, Daigoro Takahashi, Rei Okada, Yasunori Nishida, Kazuhiko Kitaguchi, Naoto Gotohda, Shinichiro Takahashi, Masaru Konishi

**Affiliations:** Division of Hepatobiliary and Pancreatic Surgery, National Cancer Center Hospital East, 6-5-1 Kashiwanoha, Kashiwa, 277-8577 Japan

**Keywords:** Mucinous cystic neoplasm of the liver, Ovarian-like stroma, Prognosis

## Abstract

A 71-year-old woman was referred to our institution for further investigation of epigastric pain. The patient had been detected to have a multilocular cyst in the medial segment of the liver measuring 69 mm in diameter at another hospital 2 years ago, and the diameter of the cyst had increased to 90 mm. Although the cyst had gradually increased in size, there was no evidence of mural nodules. As we were concerned about the malignant potential of the lesion, a left hepatic segmentectomy was performed. Pathologically, the cyst was lined by columnar and cuboidal epithelium with low-grade atypia. The epithelium covered an ovarian-like stroma, and the diagnosis was mucinous cystic neoplasm of the liver (MCN-L) with low-grade intraepithelial neoplasia. MCN-L is a rare disease and its characteristics are still poorly understood. MCN-L occurs at a lower frequency as compared to the counterpart of MCN of the pancreas, further investigations are necessary to clarify the biological malignancy of MCN-L.

## Background

Previously, mucinous cystic neoplasm of the liver (MCN-L) had been classified as biliary cystadenoma or biliary cystadenocarcinoma. However, the World Health Organization (WHO) classification of 2010 defined MCN-L as a counterpart of MCN of the pancreas (MCN-P) [1]. As for the case of MCN-P, the presence of ovarian-like stroma is required to establish the diagnosis of MCN-L. Ever since the diagnostic criteria became clear, elucidation of the characteristics of MCN-L has received much attention. However, MCN-L is a rare disease, occurring at a much lower frequency than MCN-P, and its characteristics are less well understood. We present a case of MCN-L that was treated by resection.

## Case presentation

**Figure 1:**
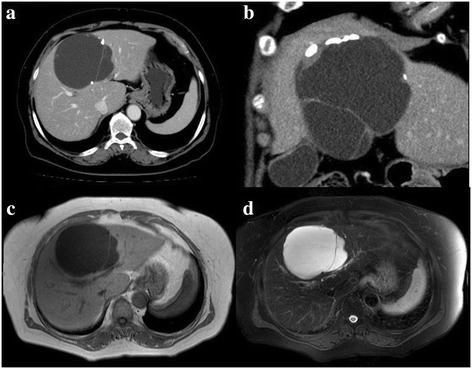
**CT and T1- and T2-weighted images.** CT showing a multilocular cystic lesion in the medial segment of the liver with calcification in the cyst wall **(a)**. Thickening of the cyst wall around the calcification **(b)**. Contrast-enhanced CT showed no enhancement of the thickened wall **(a, b)**. The cystic lesion was visualized as a hypointensity on T1-weighted images **(c)** and as a hyperintensity on the T2-weighted images **(d)**.

A 71-year-old woman visited a local hospital with the chief complaint of epigastric pain. Abdominal computed tomography (CT) revealed a large cystic mass in the liver measuring 69 mm in size, and a wait-and-watch approach was adopted. After 2 years of follow-up, the CA19-9 level, which was within normal range at first, increased to 188.5 U/ml, and CT revealed an increase in the diameter of the cystic lesion to 91 mm, and the patient was referred to our institution. At the time of her first visit to our institution, most of the laboratory data were within normal limits, and the serum CA19-9 level had increased further to 351.6 U/ml. Abdominal ultrasonography revealed a multilocular cystic lesion with a septum in the liver, measuring 110 mm in the largest diameter. CT revealed a multilocular cyst with a cyst-in-cyst appearance in the medial segment of the liver (Figure 1a). Coronal CT images revealed calcification of the cystic wall and thickening of the cystic wall around the calcification (Figure 1b). No contrast enhancement of the cystic wall or of the thickened cystic wall around the calcification was observed. No communication between the cystic lesion and the bile duct could be confirmed on CT. On magnetic resonance imaging (MRI), the cystic mass was visualized as a hypointensity on T1-weighted images (Figure 1c) and as a hyperintensity on T2-weighted images (Figure 1d).

Based on the findings that the appearance of cyst is multiloculated, there is no communication with the large bile ducts, no bile duct dilatation, and no papillary lesion in the bile ducts, the patient was diagnosed clinically as having MCN-L. There was no evidence of malignancy; however, the cystic mass had gradually increased in size and become symptomatic. Malignant transformation could not be ruled out; therefore, we performed left segmentectomy of the liver. The cystic mass was in contact with the hilar plate, but could be separated easily.

**Figure 2:**
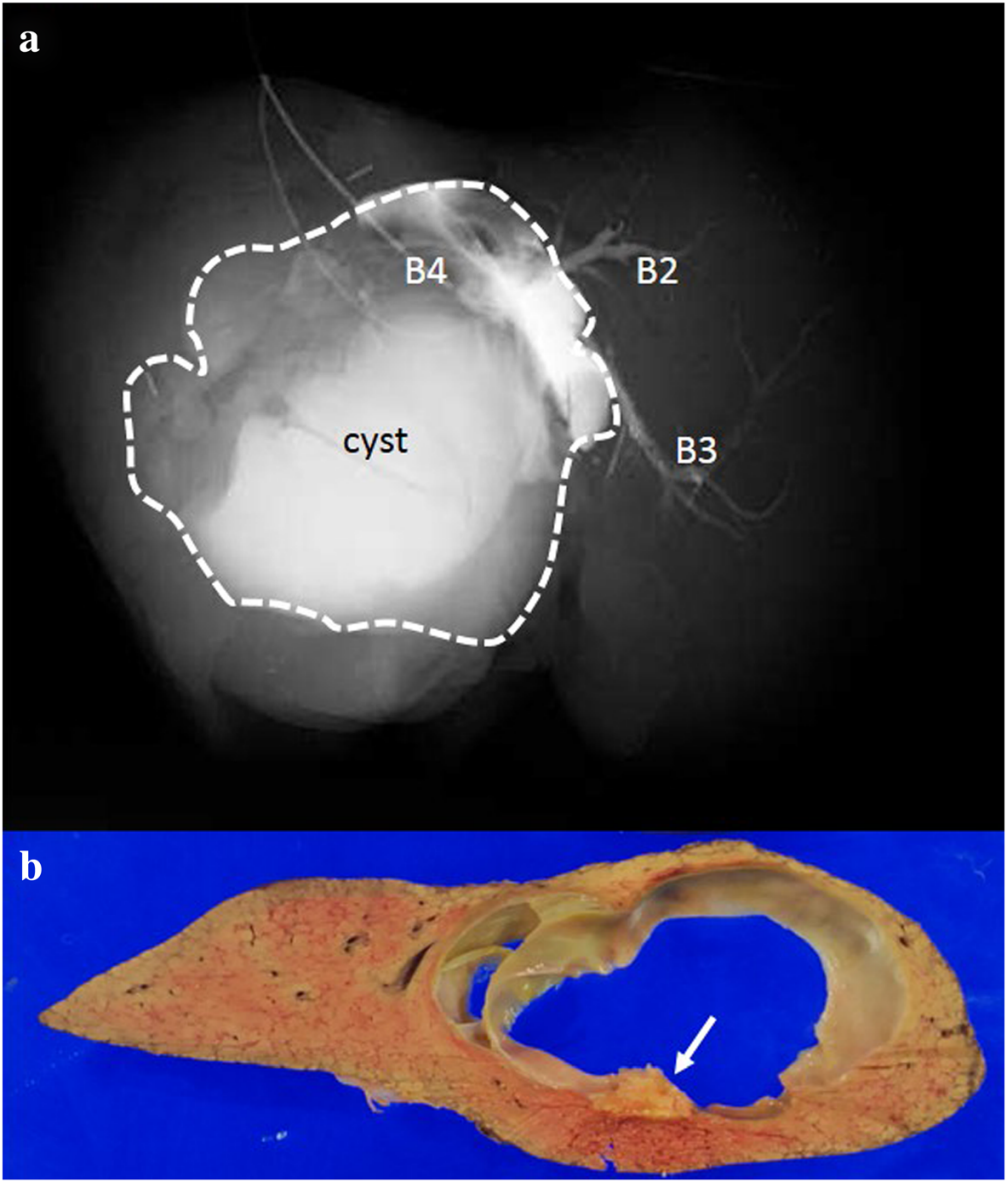
**The cyst showed contrast enhancement following postoperative injection of contrast medium into B4.** The dotted line shows an outline of the cyst **(a)**. The resected specimen was a multilocular cystic lesion covered by a thick fibrous capsule **(b)**. Calcification was found in the cyst wall (arrow). No mural nodules were found in the cystic lesion.

The cyst contents showed enhancement following postoperative injection of contrast medium into the bile duct of segment 4 (Figure 2a). The cyst contents consisted of a clear liquid of low viscosity. Sections of the specimen showed a multilocular cyst covered by a thick capsule, with calcification in the wall and thickening of the cystic wall around the calcification (Figure 2b). No mural nodules were found.

**Figure 3:**
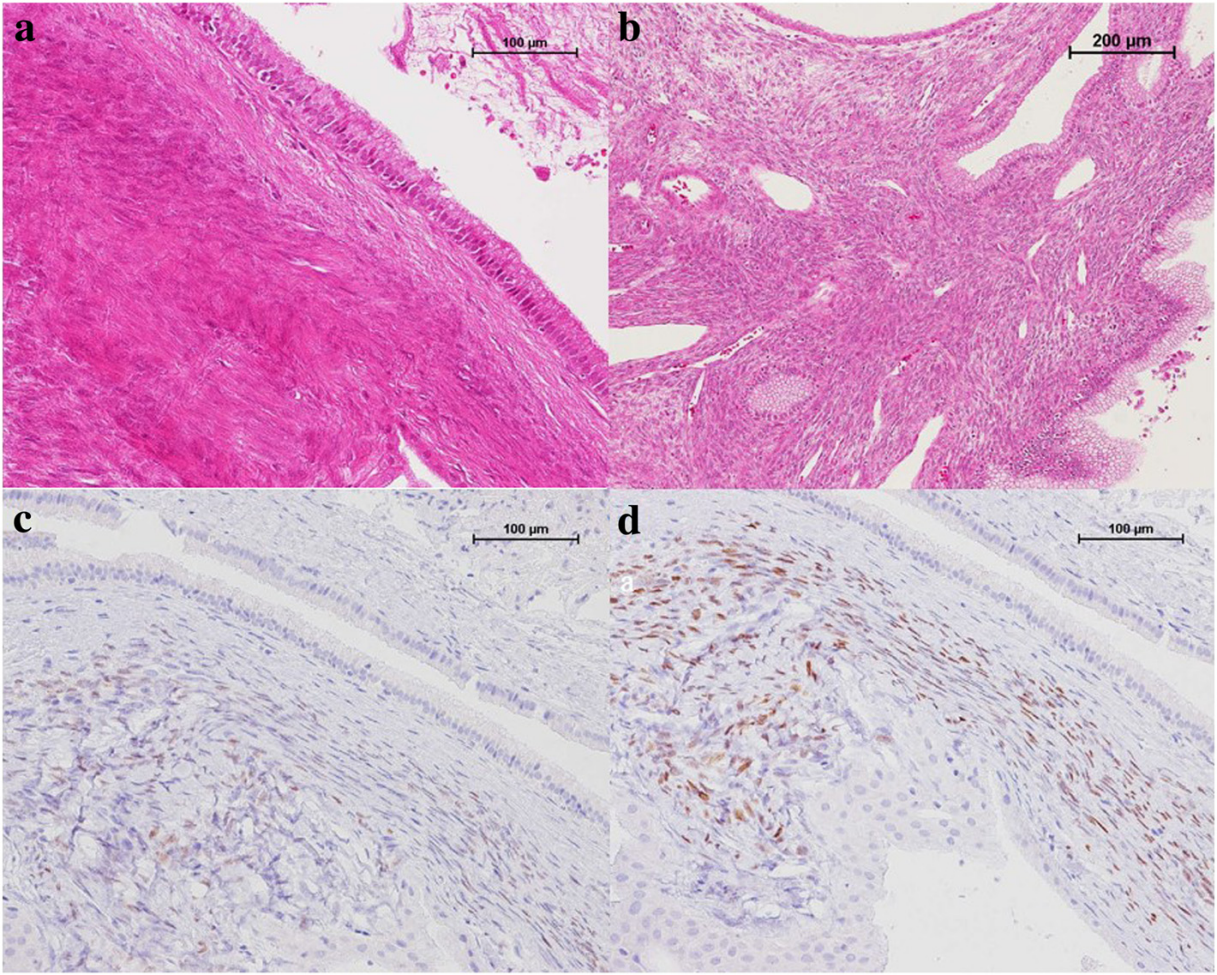
**The cyst and stromal cells.** The cyst was lined by cuboidal columnar epithelium, with low-grade atypia of the epithelial cells (**(a)** H&E staining, ×200). The columnar and cuboidal epithelium covered an ovarian-like hypercellular stroma (**(b)** H&E staining, ×100). This stroma was immunoreactive for estrogen and progesterone receptors (**(c)** estrogen receptor staining, ×200; **(d)** progesterone receptor staining, ×200).

Pathologically, the cyst was lined by columnar and cuboidal epithelium with low-grade atypia (Figure 3a). The epithelium was surrounded by an ovarian-like hypercellular stroma, and the stromal cells were spindle-shaped with oval to elongated nuclei (Figure 3b). The stromal cells were immunohistochemically positive for estrogen and progesterone receptors (Figure 3c,d). The thickened cystic wall around the calcification was composed of hyalinized tissue. The diagnosis was MCN-L with low-grade intraepithelial neoplasia according to the WHO classification of 2010.

The postoperative course was uneventful, and the patient was discharged from our institution on postoperative day 10. At present, 6 months since the surgery, the patient remains alive with no evidence of recurrence.

MCN-L, defined as a cyst-forming epithelial neoplasm of the liver, is a rare entity and is reported to account for <5% of all liver cysts [1,2]. Typical symptoms in patients with MCN-L are epigastric pain and abdominal fullness [1,3]. Similar to MCN-P, MCN-L is a multiloculated cystic tumor with septae, usually showing no communication with the bile duct, and the presence of mural nodules and papillary projections is considered to constitute evidence of malignancy [1,2,4]. The differential diagnosis of MCN-L includes intraductal papillary neoplasm of the bile duct (IPNB) and intrahepatic cholangiocarcinoma with cystic change. The characteristic findings of IPNB, including communication with the bile ducts, bile duct dilatation, and papillary projections in the bile ducts, are useful for the diagnosis of MCN-L [1,5]. However, differential diagnosis between MCN-L and cyst-forming IPNB is difficult. In such cases, the presence of ovarian-like stroma is required to establish the diagnosis of MCN-L [2,6,7]. For treatment, Zen et al. [7] reported that a large size of the cyst (>100 mm) at initial presentation, an increase in the size during follow-up, and manifestation of symptoms are indications for resection. In the event of difficulty in distinguishing between benign and malignant behavior of the lesion, complete surgical resection is critical [2].

MCN-L is defined as the pancreatic counterpart MCN-P. Shiono et al. [4] reported MCN in various organs (pancreas, liver, spleen, mesenterium), with ovarian-like stroma as the common clinicopathological feature. However, it is still unclear whether the biological characteristics of MCN-L are similar to those of MCN-P, because MCN-L is a rare entity.

We have experienced a total of four resected cases of MCN-L, including the present case, from our institution. So far, 76 cases of MCN-L, defined as a cyst-forming epithelial neoplasm with ovarian-like stroma, have been reported in all [3,6,7]. The clinicopathologoical characteristics are shown in Table 1. The similarities between our cases and the past reported cases are that MCN-L occurred predominance in middle-aged females, the malignant transformation rate was low, and the prognosis was good. Moreover, the symptom in all the 11 symptomatic cases was abdominal pain. Meanwhile, the difference among them is that two of our cases (50%) showed communication between the cyst and the bile ducts. However, the communication was not diagnosed by preoperative imaging, but by postoperative injection of contrast medium into the bile duct. Yamao et al. [8] reported that a communication between the cyst and the pancreatic duct was demonstrated in 18.1% of 156 cases of MCN-P. They explained that the communication could be due to erosion of the expanding cyst wall into the ducts to form a fistula, rather than being of true intraductal origin. The cyst diameter of MCN-L is large, and the reason for the communication between the cyst and the bile duct is as expected as in MCN-P.

**Table 1:** **Clinicopathological features of previously reported cases of MCN of the liver**

	**Zen et al.**	**Li et al.**	**Kubota et al.**	**This study**	**Total**
	**(** ***n*** **= 54)** **[** 7 **]**	**(** ***n*** **= 13)** [6]	**(** ***n*** **= 9)** **[** 3 **]**	**(** ***n*** **= 4)**	**(** ***n*** **= 80)**
Age (years), median (range)	52.5 (21 to 80)	43 (28 to 60)	65 (60 to 65)^a^	70.5 (46 to 76)	-
Gender, male:female (female %)	4:50 (92.6)	2:11 (84.6)	0:9 (100)	0:4 (100)	6:74 (92.5)
Presence of symptom, *n* (%)	-	5 (38.5)	3 (42.9)	3 (75)	11 (42.3) (*n* = 26)
Communication with duct, *n* (%)	-	0 (0)	0 (0)	2 (50)	2 (7.7) (*n* = 26)
Tumor size (mm), median (range)	100 (29 to 240)	112 ± 56^b^	70 (35 to 125)^a^	85 (30 to 120)	-
Histological type, *n* (%)					
Low or intermediate grade	53 (98.2)	6 (46.2)^c^	7 (77.8)	4 (100)	70 (87.5)
High grade	0 (0)	2 (15.4)^d^	0 (0)	0 (0)	2 (2.5)
Associated invasive carcinoma	1 (1.9)	5 (38.5)^e^	2 (22.2)	0 (0)	8 (10)
Prognosis, dead:alive	0:54	0:13	0:9	0:4	0:80

^a^Median (25th percentile, 75th percentile). ^b^Mean ± standard deviation. ^c^Adenoma. ^d^Borderline neoplasm. ^e^Carcinoma *in situ* (1) and carcinoma (4).

Comparison of the clinicopathological features between 156 cases of MCN-P, reported by the Japan Pancreas Society [8], and 80 cases of MCN-L in total, including the four cases from our institution, are shown in Table 2. The predominance in females was common to both diseases. Pathologically, the malignant transformation rate was low in both diseases, and the prognosis of both was good. However, whereas all the cases of MCN-L survived, 2.6% of patients with MCN-P died of the disease. Therefore, MCN-L may have a better prognosis than MCN-P. However, MCN-L has less or lesser number of cases than MCN-P, and more cases are needed to clarify the prognosis for patients of MCN-L.

**Table 2:** **Comparison between patients with MCN of the liver and patients with MCN of the pancreas**

	**Liver**	**Pancreas**
	**(** ***n*** **= 80)**	**(** ***n*** **= 156)** **[** 8 **]**
Gender, male: female (female %)	6:74 (92.5)	3:153 (98.1)
Presence of symptom, *n* (%)	11 (42.3) (*n* = 26)	67 (48.1)
Communication with duct, *n* (%)	2 (7.7) (*n* = 26)	25 (18.1)
Histological type, *n* (%)		
Adenoma	72 (90)^a^	129 (82.7)
Carcinoma	8 (10)^b^	27 (17.3)
Non-invasive		21 (13.4)
Minimally invasive		4 (2.6)
Invasive		2 (1.3)
Prognosis, dead:alive (dead %)	0:80 (0)	4:152 (2.6)

^a^Low- and high-grade intraepithelial neoplasia. ^b^Associated invasive carcinoma.

## Conclusions

The definition of MCN-L as a pancreatic counterpart MCN-P appears to be appropriate, because of the similarity of the clinicopathological features between MCN-L and MCN-P. Both diseases have a good prognosis. However, MCN-L is a rare disease and occurs at a lower frequency as compared to MCN-P; further investigations are necessary to clarify the biological malignancy of these tumors.

## Consent

Written informed consent was obtained from the patient for publication of this case report and any accompanying images. A copy of the written consent is available for review by the Editor-in-Chief of this journal.
